# Regulation of phage therapy medicinal products: developments, challenges, and opportunities

**DOI:** 10.3389/fcimb.2025.1631359

**Published:** 2025-10-29

**Authors:** Miriam Fuerst-Wilmes, Vanessa Respondek, Michael Schramm, Nils Lilienthal, Katrin Buss, Anja Duechting

**Affiliations:** Federal Institute for Drugs and Medical Devices, Bonn, Germany

**Keywords:** bacteriophages, phage therapy medicinal products, regulatory advice, antimicrobial resistance, antibacterial treatment, non-traditional product

## Abstract

Due to their biological properties, bacteriophages represent a regulatory specialty and, at the same time, a challenge with regard to medicinal product approval. Established European guidelines on pharmaceutical quality, preclinical development, and clinical development are only partially applicable. The growing threat posed by infections with multidrug-resistant bacteria has not only boosted the development of bacteriophages for the treatment of bacterial infections in recent years but has also led to substantial progress in adapting regulatory requirements. In 2024, harmonized quality criteria for phage therapy medicinal products and active substances were implemented for the first time in the European Pharmacopoeia. Future European pharmaceutical legislation and recent national acts such as the German Medical Research Act are intended to enable exemptions that address the specific characteristics of phage therapeutics and open new regulatory pathways. Increasing amounts of data on clinical use of phage therapeutics are being published; however, the anticipated breakthrough in the form of a demonstration of efficacy in randomized controlled clinical trials has not yet been achieved. Growing experience with innovative phage preparations has been utilized to adjust regulatory requirements. On the path to approval of a defined phage therapy medicinal product, the evidence-based demonstration of efficacy and safety in randomized controlled clinical trials is the next and decisive step.

## Introduction

Bacteriophages (phages, for short) are one of the most promising tools in combating infections caused by multidrug-resistant bacteria. Although the first (documented) clinical applications of phage preparations date back nearly 100 years and they have been continuously used in Eastern Europe to this day, there is still a lack of established standards for defining the pharmaceutical quality, safety, and efficacy of these preparations (Summers, 1999; Strathdee et al., 2023; Palma and Qi, 2024). In response to the growing threat posed by multidrug-resistant bacteria, numerous funding initiatives have been launched in recent years which have spurred the development of bacteriophages as medicinal products (2025). One of the challenges has always been to incorporate phage therapy medicinal products (PTMPs), with their unique biological properties and modes of action, into the existing regulatory framework or to adapt the latter accordingly. Requirements for the quality, safety, and efficacy of phage preparations have evolved in recent years both on a national level in Germany and across Europe. This article aims to provide an overview of these developments and to depict the current state of regulatory requirements for PTMPs in Europe and, in particular, in Germany.

## Quality aspects

### Manufacture and application of PTMPs

In Europe, bacteriophages for therapeutic use in humans are considered biological medicinal products pursuant to Article 1 (2) and (3) in conjunction with Annex I, Part 1, 3.2.1.1 (b) Sentences 3 and 4 of Directive 2001/83/EC). In Germany, they are classified as medicinal products by function pursuant to Section 2 (1) in conjunction with Section 3 No. 4 of the Medicinal Products Act (*Arzneimittelgesetz*, AMG). Prior to use in patients, they generally require either a marketing authorization or an authorization for use as investigational medicinal product in a clinical trial.

Currently, PTMPs are manufactured and applied via two distinct pathways:

1. Standardized phage preparations (often mixtures of various phages) are produced industrially in advance and used in the context of a clinical trial. These medicinal products are subject to the obligation to obtain a marketing authorization and manufacturing authorization. They can, in principle, be assessed for their quality, efficacy, and safety through clinical trials and ultimately be approved under the existing regulatory pathways. As authorized medicinal products, they would then be available to all patients in accordance with the approved indication. However, current regulatory requirements—for example, for changes in the composition of approved PTMPs—present challenges for these products (e.g., replacement or adaptation of phages in the medicinal product due to resistance development of the bacterial pathogens – “moving targets”). Future European pharmaceutical legislation could establish more suitable structures and regulatory concepts in this regard.
2. Phage preparations are manufactured as individual magistral formulations (referred to as *formula magistralis* in Directive 2001/83/EC) specifically for the individual patient based on a physician’s prescription, usually in a (hospital) pharmacy, shortly before application. These products are exempted from the obligations to obtain a marketing and manufacturing authorization, as they do not fall within the scope of Directive 2001/83/EC pursuant to Article 3 (1). In Germany, the obligation to obtain a marketing authorization under Section 21 (1) AMG applies to finished medicinal products (as defined in Section 4 (1) Sentence 1 AMG), but not to individual magistral formulations, as these are not manufactured “beforehand” according to the definition in Section 4 (1) No. 1 AMG, but rather “in the pharmacy in particular cases on the basis of a prescription or other request by an individual person (…),” as specified in Section 1a (8) of the Ordinance on the Operation of Pharmacies (*Apothekenbetriebsordnung*, ApBetrO). Use of phage preparations typically occurs under the conditions of an attempt to restore health or alleviate suffering for an individual patient (cf. Section 37 of the Declaration of Helsinki). Modifications to PTMPs exempted from the obligation to obtain a marketing authorization can be implemented more flexibly and quickly, since regulatory steps for approval of the changes are not required. However, this “last-resort” treatment is only available to a small number of patients in exceptional cases.

## Quality of PTMPs

Pursuant to Section 55 (8) AMG, all medicinal products must be manufactured in accordance with recognized pharmaceutical rules. In accordance with Section 55 (1) AMG, these rules are laid down in the German pharmacopoeia. They include, among others, the quality of medicinal products and the substances used in their manufacture. The rules of the pharmacopoeia provide recognized common standards for the quality of medicinal products and their components, thereby ensuring patient safety. In March 2024, during its 178^th^ session, the European Pharmacopoeia Commission adopted the general chapter “Phage therapy medicinal products (5.31)”. The chapter was published in July 2024 in Supplement 11.6 of the European Pharmacopoeia (Ph. Eur.) and came into force in January 2025. This chapter becomes legally binding once it is referenced in a monograph (or in another general chapter that is in turn referenced in a monograph). For the first time, this chapter defines quality criteria for PTMPs and active substances that are harmonized across Europe. It encompasses both human and veterinary medicinal products subject to as well as exempt from the obligation to obtain a marketing authorization. In order to accommodate the wide range of highly diverse products, the chapter establishes fundamental requirements while allowing sufficient flexibility.

This framework of requirements addresses, among others:

- Specifications for bacterial and phage banks, including critical quality attributes (e.g. identity, purity, activity).
- Production process and purification steps, including in-process controls.
- Specifications for active substances and drug products, including relevant quality attributes (e.g. identity, microbial quality, activity).

A critical quality attribute of all PTMPs is the biological activity, which is typically determined using a plaque assay. However, this method has not yet been standardized. At its 176^th^ session in June 2023, the European Pharmacopoeia Commission decided to draft a new general chapter (2.7.38) on the determination of bacteriophage potency, with the aim of providing harmonized provisions for the conduct, standardization, and validation of the assay. A draft version of this chapter was published for public consultation in April 2025.

To address the requirements for PTMPs subject to the obligation to obtain a marketing authorization, the European Medicines Agency (EMA) has issued specific guidelines. For veterinary medicinal products, the “Guideline on quality, safety and efficacy of veterinary medicinal products specifically designed for phage therapy” (EMA/CVMP/NTWP/32862/2022) has been published. A corresponding guideline for human phage therapeutics is currently under development (“Guideline on quality aspects of phage therapy medicinal products”, EMA/CHMP/BWP/1/2024). Relevant aspects (also considering Ph. Eur. Chapter 5.31) of this latter guideline include, among others:

- Quality of starting materials, excipients, and active substances.
- Manufacture and specifications of bacterial and phage banks.
- Characterization of the active substance and its impurities (e.g., endotoxins).
- Manufacturing process, including process development, validation, and controls.
- Specifications of the active substance and finished product, including analytical and validation.
- Reference standards and stability.

Publication of the draft guideline for public consultation is scheduled for November 2025. In addition, the principles of existing EMA and ICH guidelines for biotech products are, where applicable, also relevant for PTMPs. These include, for example, guidelines on the characterization of cell banks (ICH Q5D), stability (ICH Q5C), specifications (ICH Q6B), analytical methods (ICH Q2, Q14), as well as further topics (ICH Q8 – Q11). Particularly relevant for phage products as investigational medicinal products in clinical trials is the “Guideline on the requirements for quality documentation concerning biological investigational medicinal products in clinical trials” (EMA/CHMP/BWP/534898/2008 Rev. 2).

Furthermore, a S2k-guideline is currently being developed by the Association of the Scientific Medical Societies in Germany (*Arbeitsgemeinschaft der Wissenschaftlichen Medizinischen Fachgesellschaften*, AWMF), with finalization planned for December 2025. This guideline will provide recommendations on the personalized manufacture and therapeutic application of bacteriophages and address legal and logistical considerations specific to Germany. Through its consensus-based development process and the involvement of several medical societies, the guideline will yield a harmonized set of recommendations of varying evidence levels, providing guidance and a framework for, among others, the manufacture of personalized PTMPs, including their quality attributes and quality control measures.

The multitude of ongoing activities in the area of the quality and manufacturing of PTMPs underscores the potential of these products, even though clinical efficacy has not yet been conclusively demonstrated in randomized controlled clinical trials (RCTs). Nonetheless, systematic case observations increasingly suggest that phage therapy (most likely in combination with antibiotics) may represent a viable treatment option for infections caused by antibiotic-resistant bacteria (Pirnay et al., 2024). The above-described regulatory developments have introduced and continue to establish specific requirements for this special class of medicinal products, with the aim of ensuring a consistently high and harmonized quality across Europe—ultimately in the interest of patient safety.

## Manufacturing authorization and good manufacturing practice

In principle, the manufacture of medicinal products on a commercial basis in Europe is subject to an obligation to obtain a manufacturing authorization (Art. 40 of Directive 2001/83/EC; Section 13 (1) AMG) and requires compliance with the principles and guidelines of Good Manufacturing Practice (GMP). This compliance is verified as part of the procedure for granting a manufacturing authorization (Section 1 (1) in conjunction with Section 2 (3) and Section 13 (1) of the Ordinance on the Manufacture of Medicinal Products and Active Substances (*Arzneimittel- und Wirkstoffherstellungsverordnung*, AMWHV)).

The personalized manufacture of medicinal products in a pharmacy, within the ordinary course of pharmacy operations, based on a medical prescription for a specific patient is exempted from the obligation to obtain a manufacturing authorization (Section 13 (2) sentence 1 AMG). However, also in this case, manufacture must conform to the recognized pharmaceutical rules (Section 55 (8) AMG), and the quality of both starting materials and the finished product must be ensured (Section 6 (1) in conjunction with Section 11 of the ApBetrO). The decision whether the preparation of individualized PTMPs constitutes the “ordinary course of pharmacy operations” lies within the responsibility of the competent authority (in Germany, that of the federal state in which the pharmacy is located). If this classification is not granted, the preparation is subject to the obligation to obtain a manufacturing authorization pursuant to Section 13 (1) AMG.

Personalized preparation of PTMPs may, in principle, also be carried out by physicians (“directly under his/her professional responsibility for personal use by a specific patient”; Section 13 (2b) sentence 1 AMG). If a GMP-compliant phage active substance is used for PTMP preparation, this activity is exempted from the obligation to obtain a manufacturing authorization under Section 13 (2b) AMG (although it must be notified under Section 67 (2) AMG). However, the preparation of a phage active substance by a physician in order to manufacture a PTMP is subject to the obligation to obtain a manufacturing authorization pursuant to Section 13 (1) sentence 3 AMG.

The manufacture of genetically modified or recombinantly produced PTMPs—defined as Advanced Therapy Medicinal Products (ATMPs)—is in any case subject to the obligation to obtain a manufacturing authorization (Section 13 (1) AMG). Furthermore, the provisions of directives 2001/18/EC and 2009/41/EC should be taken into account for genetically modified phages, as applicable.

With the enactment of the Medical Research Act (*Medizinforschungsgeset*z), which amends both the AMG and the AMWHV accordingly, the specific requirements of individualized phage therapeutics are taken into account. Under the newly introduced Section 14 (6) AMG, “the competent higher federal authority can publish recommendations on the interpretation of the principles and guidelines of good manufacturing practice.” Furthermore, pursuant to the new Section 14 (7) AMG, on application by a competent authority, the competent higher federal authority may prepare and publish “an opinion on the interpretation of the principles and guidelines of good manufacturing practice for the medicinal products”. The AMWHV has been amended accordingly in Section 3 (2) by reference to Section 14 (6) AMG.

These regulatory amendments allow for individual and risk-based adaptations of the general GMP requirements to the specific circumstances of PTMP manufacture, while ensuring consistent high quality and safety of these products. This enables the GMP framework to better account for the particular characteristics of phages as medicinal products. Publication of the recommendations pursuant to Section 14 (6) AMG is planned for end of 2025. These recommendations are not legally binding for the competent authorities, but any deviation from them should be justified.

## Outlook on regulation of PTMPs

The current regulatory framework cannot fully accommodate the specific requirements of PTMPs. Challenges arise particularly in relation to marketing authorization or subsequent modification of PTMPs in order to introduce new or replace existing phage(-strains) in response to resistance development, without requiring a separate marketing authorization for each (new) phage. The use of a platform technology and the possibility of authorizing PTMPs with (partially) variable composition could provide a viable solution by enabling regulatory grouping of the manufacture (including manufacturing authorization) and approval of various phage(-strains).

In the adopted proposals for a new Directive and a new Regulation (cf. documents 2023/0132 (COD) and 2023/0131 (COD)), which revise and replace the existing general pharmaceutical legislation (Directive 2001/83/EC and Regulation (EC) No. 726/2004), the European Commission addresses PTMPs (subject to the obligation to obtain a marketing authorization), as well as the aforementioned regulatory challenges(2025). The proposals introduce new concepts such as “adapted frameworks” (Article 28 of the proposal for a new EU Directive) and a “regulatory sandbox” (Articles 113–115 of the proposed new EU Regulation), which provide for “adapted, enhanced, waived or deferred requirements”. Moreover as forward looking legal frameworks, they establish new pathways for the approval of medicinal products “comprised of a fixed component and a variable component that is pre-defined in order to, where appropriate, target different variants of an infectious agent (‘platform technology’)” (Article 15 of the proposed new EU Directive). These new and flexible regulatory mechanisms could prove highly beneficial, enabling a pragmatic approach to both initial marketing authorization and subsequent modifications of the products, thereby granting all patients access to PTMPs as a therapeutic option.

## Preclinical aspects

The following outlines the assumptions underlying the considerations for the preclinical development program: Bacteriophages are biologicals that do not replicate in eukaryotic cells and exert no direct pharmacological effects within them. Therefore, virulent phages are considered non-hazardous (non-toxic) to humans. Both animals and humans are constantly and naturally exposed to large quantities of bacteriophages (Clokie et al., 2011; Salmond and Fineran, 2015).

Sections of the “Guideline on the evaluation of medicinal products indicated for treatment of bacterial infections” (CPMP/EWP/558/95 Rev. 3) can be applied to PTMPs (Section 4.1: Non-clinical assessment of anti-bacterial activity), and the guideline for veterinary phage products (EMA/CVMP/NTWP/32862/2022) also includes guidance relevant to the preclinical program. While currently no specific guideline exists for the development of PTMPs for human use—and hence no dedicated description of preclinical requirements, the aforementioned guidelines provide an initial orientation regarding regulatory expectations. It is widely acknowledged that regulatory requirements for new medicinal products should be proportionate to the risks associated with their intended use. Based on this principle, deviations from or reductions in the standardized set of preclinical investigations expected for newly developed medicinal products—such as *pharmacology, pharmacodynamics, pharmacokinetics (PK), repeated-dose toxicity, genotoxicity, carcinogenicity, reproductive toxicity*, and other toxicological endpoints like *local tolerance*—can be scientifically justified. Nevertheless, applicants should address the safety of the selected phages (and the final lots/batches) in terms of toxicity endpoints and support the non-clinical dossier of a marketing authorization application with corresponding information and discussions.

Potential safety concerns may arise from the presence of microbiological contaminants, such as endotoxins and other potent pro-inflammatory substances released as a consequence of bacterial lysis during phage propagation (Liu et al., 2021). Endotoxins and exotoxins as potential microbiological contaminants are critical quality attributes of the product. These and other bacterial components have to be controlled in phage products. Current experience in this area indicates that product quality (purity) is a key determinant of safety (see section Quality Aspects).

*In vitro* susceptibility studies should demonstrate efficacy of the phages against the targeted bacterial pathogens. Such studies should be conducted under conditions that closely resemble those at the site of infection (e.g., presence of a biofilm). When using phage combinations, compatibility among the phages with respect to a representative clinical isolate has to be ensured. Even though conventional studies of absorption, distribution, metabolism, and excretion (PK) are considered unsuitable for phage products, the applicant should investigate or discuss—based also on literature data—the absorption at the site of administration, distribution, and expected degradation pathways of the phages. Depending on the intended route of administration (e.g., inhalational or intravenous), different PK data are required.

Due to antibiotic resistance, antibiotics are sometimes investigated in combination with phages. Potential antagonistic or synergistic interactions between phages and antibiotics should be considered in the development of a PTMP. Reports indicate that the outcome of phage–antibiotic interactions depends on several factors, including the class of antibiotic, type of phage, and pairing stoichiometry (Gu Liu et al., 2020). Additionally, resistance mechanisms and phage-induced immune responses may be relevant for phage monotherapy and/or phage–antibiotic combination therapy (Krut and Bekeredjian-Ding, 2018; Doub, 2020). These aspects should also be discussed or explored early in the development process.

Regarding the above aspects, there is currently no requirement for conducting *in vivo* animal studies. Rather, it is the responsibility of the applicant to demonstrate the efficacy of the selected phages against the target bacteria, to establish the safety of the phage product, and to determine the safe and effective dose in humans.

The Federal Institute for Drugs and Medical Devices *(Bundesinstitut für Arzneimittel und Medizinprodukte*, BfArM), as the competent authority for (non-genetically modified) phages in Germany, does not expect stand-alone studies on reproductive and developmental toxicity for PTMPs. It is assumed that virulent bacteriophages do not interact directly with eucaryotic DNA or other chromosomal material. Consequently, the standard test battery to assess genotoxicity and carcinogenicity studies may also be omitted.

In conclusion, an effectively designed preclinical program for phage PTMPs can meet the regulatory requirements for safe drug development when targeted adaptations and exceptions are scientifically justified.

## Clinical aspects

Phages have been used to treat bacterial infections for more than 100 years. However, evidence-based proof of the efficacy of phage therapy based on a sufficiently large and well-designed RCT (conducted in line with current standards), which would be a prerequisite for marketing authorization of a PTMP, is still lacking. From a regulatory perspective, efficacy data from Phase 2 and Phase 3 RCTs are urgently needed in order to establish phage therapy as an effective therapeutic option and to adapt regulatory requirements. Data on the efficacy and safety of phages originate primarily from individual applications in patients with limited treatment options and uncontrolled studies. In the few RCTs conducted and published so far that included at least 20 patients (only Phase 1/2 and Phase 2 studies; see **Table 1**), in most cases, the efficacy of phage therapy could not be sufficiently demonstrated (Sarker et al., 2016; Jault et al., 2019; Leitner et al., 2021).

**Table 1:** Examples of published randomized controlled clinical trials (phase 1/2 and 2 with n ≥20) in which, among other things, the efficacy of phages was investigated (as of 10/2025)*.

Study	Study description	Included patients	Phage product/application/dosage	Result in terms of efficacy	Reference
Phase 1/2	Randomized, placebo-controlled, double-blind study in adult patients with chronic otitis media caused by *Pseudomonas aeruginosa*	n = 24	Fixed cocktail of 6 phages; topical application; Single dose of 6x 10^5^ PFU	• Significant clinical improvement • Reduction of bacterial load • Replication of the applied phages	(Wright et al., 2009)
	Randomized, placebo-controlled study in hospitalized children (6–24 months of age) with acute bacterial diarrhea caused by *Escherichia coli*	n = 120	Fixed cocktail of 11 phages or commercial product from Russia (of at least 17 phages); oral application; 3.6x 10^8^ PFU or 1.4x 10^9^ PFU over 4 days	• No clinical improvement • No replication of phages in the intestine Reasons for negative study result: • only 60% of the included patients had a proven *E. coli* infection • Insufficient coverage of the phage cocktail • Lower *E. coli* titer than expected • pH stability of the phage cocktail unclear	(Sarker et al., 2016)
	Randomized, placebo-controlled, double-blind, multicenter study in adult patients with *Pseudomonas aeruginosa-*infected burns (PhagoBurn)	n = 27	Fixed cocktail of 12 phages; topical application; 1x 10^6^ PFU/ml for 7 days	• Study was terminated early due to insufficient recruitment and lack of efficacy Reasons for negative study result: • Instability of the phage cocktail and therefore too low phage concentration applied	(Jault et al., 2019)
	Randomized, placebo-controlled, double-blind monocenter study in adults with chronic wound infections (mono- and polybacterial infections)	n = 60	Individualized phage cocktail; topical application, 0.5x 10^9^ PFU on alternate days for up to 3 months in addition to standard of care	• Improvement of various wound parameters over time • 93.3% of the wounds became sterile in 39 days (median sterility time), followed by complete healing by the end of 90 days	(Karn et al., 2024)
Phase 2	Randomized, placebo-controlled, double-blind study for the treatment of urinary tract infections (caused by *Enterococcus* spp., *E. coli*, *Proteus mirabilis*, *P. aeruginosa*, *Staphylococcus* spp., *Streptococcus* spp.) in men with planned transurethral resection of the prostate	n= 113	Commercial phage cocktail from Georgia; topical application (intravesical); 2x daily for 7 days	• No difference in efficacy between the treatment arms Reasons for negative study result: • Indication too broad • High spontaneous healing rate for uncomplicated urinary tract infections	(Leitner et al., 2021)

*Further published RCTs (e.g. the recently published study by Weiner et al (Weiner et al., 2025)) are not included in the Table because of the small number of patients recruited and hence very limited informative value in the present context.

The results of these studies (negative and positive) should be considered when planning further studies (phase 2 and phase 3) in order to provide the outstanding proof of efficacy of phage therapy.

In a recently published retrospective observational study by Pirnay et al (Pirnay et al., 2024), 100 cases of personalized phage treatment were described. Personalized phage treatment led to clinical improvement in 77% of cases and to eradication of targeted bacteria in 61% of cases. However, the quality of evidence cannot be considered similar to that of a RCT as there was no control group and the efficacy (and safety) of the phage application was not assessed on the basis of objective, predefined criteria but only by the physician´s assessment. In addition, phages were applied for various indications (in 70% of cases in combination with an antibiotic) and in different ways leading to very small numbers of treated patients per indication and method of administration meaning that these study results do not have any statistical power. Nevertheless, the experience gained from these and other personalized phage applications can be incorporated into the design of RCTs (e.g., with regard to phage dose, method of administration and duration of treatment).

At present, there are no specific guidelines for the clinical development program of PTMPs for human use. However, the “Guideline on the evaluation of medicinal products indicated for treatment of bacterial infections” (CPMP/EWP/558/95 Rev 3) is largely applicable (e.g., Section 5: General considerations for clinical programs).

Based on experience from published RCTs and scientific advice procedures at German national and EU level, the following aspects should be considered from a regulatory perspective for the planning of Phase 2 and Phase 3 studies and the demonstration of efficacy of PTMPs:

## Indication

Phage preparations could be used for both treatment of bacterial infections and prevention (by eradication of a potential pathogen in a specific body region). However, the phage-host specificity excludes a broad indication as many authorized antibiotics have. Particularly suitable for a proof-of-concept (PoC) study (Phase 2) are (acute) infections that are caused exclusively or predominantly by a single bacterial species (monobacterial infections) and for which phages can be applied locally.

The study design of a pivotal study (Phase 3) primarily depends on whether the phage product is intended to be used alone or in combination with antibiotics. Particularly in cases of severe and/or chronic infections, a combination therapy of phages and antibiotics could be reasonable. In this case, superiority of the combination therapy over the standard of care has to be demonstrated. For less severe infections, non-inferiority of the PTMP to standard antibiotic therapy (or placebo, if no standard of care exists) should be demonstrated.

## Dosage and method of administration

For a successful therapy, a sufficiently high number of phages has to reach the site of infection. The replication of the phages with subsequent lysis of the target bacteria is key in the so-called “active therapy” (Payne et al., 2000). However, the pharmacokinetics of phages are still insufficiently understood, and it remains unresolved whether a high concentration always correlates with a better therapeutic outcome. Furthermore, phage replication depends on the bacterial load at the site of infection (Danis-Wlodarczyk et al., 2021; Nang et al., 2023; Pirnay et al., 2024).

Nevertheless, local applications (e.g., topical or inhalative) can achieve higher phage concentrations at the infection site. Experience from personalized phage applications particularly in regard to phage concentration, dosing interval, and duration of therapy should be incorporated into the design of RCTs. Moreover, it is highly recommended to test phage replication at the infection site in a RCT.

## Diagnostics

In contrast to most antibiotics, phages are highly specific for a single bacterial species, meaning that the causative pathogen must be identified before the start of phage therapy. Thus, adequate microbiological diagnostics are required.

We highly recommend susceptibility testing prior to patient inclusion in a clinical study, although it is not necessarily required (e.g., in the case of a fixed phage cocktail with a high coverage rate). However, the susceptibility of the bacterial isolate to individual phages or to the phage cocktail should definitely be tested during the clinical study in order to enable correlation of the results of the susceptibility test with therapeutic outcome. In a pivotal study, the primary analysis should be based on all patients with phage-susceptible bacteria (“microbiological Intention-To-Treat population”; CPMP/EWP/558/95 Rev 3). Susceptibility testing at certain time points of the clinical study can provide information on potential resistance development and, depending on the study design, can also be used to adjust the treatment.

## Endpoints

For a PoC study, a microbiological primary endpoint (e.g., reduction of bacterial load) can be selected as a surrogate parameter for efficacy. However, it is recommended that, already in a PoC study, clinical endpoints are investigated to allow estimations of the clinical benefit of phage therapy. Relevant clinical endpoints could be time to clinical improvement of symptoms, duration of hospitalization, need for (or duration of) additional antibiotic therapy, frequency of reinfections, or infection rate (in case of prophylactic application).

## Patient population

The patient population to be included in a study should be clearly defined in line with the intended indication. For this, the prevalence of a specific pathogen in a given indication should be taken into account in order to be able to recruit a sufficiently large number of patients. Furthermore, based on the potential risk of phage therapy, it should be considered which patients would most likely benefit from phage therapy and in which patient population efficacy can likely be demonstrated (benefit-risk analysis).

In general, a favorable safety profile has been described for the use of phages in clinical studies (Rhoads et al., 2009; Wright et al., 2009; Sarker et al., 2016; Jault et al., 2019) as well as in personalized phage applications in patients with limited treatment options (Uyttebroek et al., 2022; Pirnay et al., 2024). In a systematic review published in 2022 comprising 52 studies on the safety and efficacy of phage therapy in difficult-to-treat infections, adverse events were reported in 33 (7%) of 441 patients receiving phage therapy and in 37 (15%) of 249 patients in the control group (Uyttebroek et al., 2022). In general, these adverse events were mild in nature and resolved after discontinuation of phage therapy. Since phage therapy appears to be safe and well tolerated, it may represent an advantage over antibiotic therapy (in terms of benefit-risk-ratio), especially for those antibiotics associated with severe side effects. In the long term, a systematic investigation of efficacy in specific patient populations, such as the pediatric population or immunosuppressed patients, would also be desirable.

## Further aspects

Neutralization of phages by antibodies has already been described in the literature and may dependent on the duration of therapy, the immune status of the patient, and the route of administration (e.g., intravenous administration) (Dedrick et al., 2021; Nang et al., 2023; Pirnay et al., 2024). Additional data on neutralizing antibodies and the correlation between antibody detection and efficacy are needed and should be systematically studied in clinical trials. To enhance/maintain therapeutic efficacy, adaptation of the phages in the medicinal product to the patient’s bacterial isolate is conceivable, e.g., in the form of so-called “phage training” or by means of genetic/synthetic modification of the phages (Bleriot et al., 2024; Ngiam et al., 2024).

## Conclusion

With regard to the regulation of phage therapeutics, various developments have recently taken place. The publication of guidelines for harmonized quality criteria for PTMPs at the European level and the adaptation of German national and European pharmaceutical legislation to the specific characteristics of phage therapeutics show how dynamic this field currently is and that important hurdles have been overcome. However, the overall aim of the marketing authorization of a first PTMP has not yet been achieved. Crucial to this will be learning from the mistakes of previously conducted clinical studies in order to design high-quality clinical studies capable of demonstrating the efficacy and safety of defined PTMPs. Regular discussions with regulators (e.g. through scientific advice meetings) are highly recommended to streamline PTMP development in accordance with regulatory requirements.
